# Phospholipase A_2_ in skin biology: new insights from gene-manipulated mice and lipidomics

**DOI:** 10.1186/s41232-018-0089-2

**Published:** 2018-12-07

**Authors:** Makoto Murakami, Kei Yamamoto, Yoshitaka Taketomi

**Affiliations:** 1 Laboratory of Microenvironmental and Metabolic Health Science, Center for Disease Biology and Integrative Medicine, Graduate School of Medicine, The University of Tokyo, 7-3-1 Hongo, Bunkyo-ku, Tokyo, 113-8655 Japan; 2 AMED-CREST, Japan Agency for Medical Research and Development, 1-7-1 Otemachi, Chiyoda-ku, Tokyo, 100-0004 Japan; 3 PRIME, Japan Agency for Medical Research and Development, 1-7-1 Otemachi, Chiyoda-ku, Tokyo, 100-0004 Japan; 4 Division of Bioscience and Bioindustry, Graduate School of Technology, Industrial and Social Sciences, Tokushima University, Tokushima, 770-8513 Japan

**Keywords:** Knockout mouse, Lipid mediator, Lipidomics, Phospholipase A_2_, Skin

## Abstract

The skin represents one of the tissues that are most profoundly influenced by alterations in the quality of lipids (lipoquality). Lipids not only constitute cellular membranes, but also serve as bioactive lipid mediators and essential components of the skin barrier. Phospholipase A_2_ (PLA_2_) enzymes supply fatty acids and lysophospholipids from membrane phospholipids, thereby variably affecting cutaneous homeostasis. Accordingly, perturbation of particular PLA_2_-driven lipid pathways can be linked to various forms of skin disease. In this review article, we highlight the roles of several PLA_2_ subtypes in cutaneous pathophysiology, as revealed by transgenic/knockout studies in combination with comprehensive lipidomics. We focus mainly on secreted PLA_2_ group IIF (sPLA_2_-IIF), which is associated with epidermal hyperplasia through mobilization of a unique lipid metabolite. We also address the distinct roles of sPLA_2_-IIE in hair follicles and sPLA_2_-IID in lymphoid immune cells that secondarily affect cutaneous inflammation, and provide some insights into species differences in sPLA_2_s. Additionally, we briefly overview the patatin-like phospholipase PNPLA1, which belongs to the Ca^2+^-independent PLA_2_ (iPLA_2_) family, as a key regulator of skin barrier function through catalysis of a unique non-PLA_2_ reaction. These knowledges on lipid metabolism driven by various PLA_2_ subtypes will open novel opportunities for translated studies toward diagnosis and therapy of human skin diseases.

## Background

The skin consists of the outer epidermis, beneath which are the dermis and subcutaneous tissue. Epidermal keratinocytes undergo a tightly regulated program of proliferation and differentiation leading to formation of the stratified epidermis, which consists of four layers including the stratum basale (SB), the stratum spinosum (SS), the stratum granulosum (SG), and the stratum corneum (SC) from the inside to the outside. For survival in a dry terrestrial environment, the epidermis constitutes a life-sustaining skin barrier, which not only prevents water loss (inside-out barrier), but also protects against invasion of environmental substances or microorganisms (outside-in barrier) [1]. In the uppermost SC, corneocytes are embedded in a lipid-rich extracellular matrix that forms lamellar membranes composed of ceramides, cholesterol, and fatty acids in a mildly acidic environment [2]. The epidermis also has immunologic functions, protecting the skin from ultraviolet damage via pigmentation of melanocytes and from external harmful stimuli by releasing various bioactive factors such as cytokines, chemokines, DAMPs (danger-associated molecular patterns), and lipid mediators, which relay signals to specialized immune cells residing in the epidermis and dermis [3].

Another important component of the skin is the hair follicle, whose morphogenesis is regulated by interactions between epidermal keratinocytes committed to hair follicle differentiation and dermal fibroblasts committed to formation of the dermal papilla of developing hair follicles [4]. These epithelial-mesenchymal interactions culminate in the formation of the hair shaft, which is surrounded by the multilayered inner root sheath and outer root sheath, the latter comprising an outermost concentric layer of keratinocytes. Hair follicles undergo repeated cycles of growth (anagen), regression (catagen), and rest (telogen) during their life span, representing one of the most regenerative organs in the body. Within the apex of the follicle are sebaceous glands, which produce sebum. The adipocyte layer within the hypodermis also constitutes a significant compartment of the skin, contributing to hair follicle activation [5], skin regeneration [6], and cold-induced adaptive thermogenesis [7].

Lipids play fundamental roles in skin physiology and pathology. Dysregulated production of polyunsaturated fatty acid (PUFA)- or lysophospholipid-derived lipid mediators can be linked to skin disorders including alopecia, inflammation, and cancer. For instance, arachidonic acid (AA; ω6 C20:4)-derived lipid mediators such as prostaglandins (PGs) and leukotrienes (LTs) have diverse roles in immune responses and keratinocyte activation [8, 9], eicosapentaenoic acid (EPA; ω3 C20:5)- or docosahexaenoic acid (DHA; ω3 C22:6)-derived resolvins attenuate skin immune responses [10, 11], and lysophosphatidic acid (LPA) controls hair homeostasis [12, 13]. Apart from these signaling lipids, linoleic acid (LA; ω6 18:2), by far the most abundant PUFA in the epidermis, is esterified to the ω-hydroxyl group of ultra-long chain fatty acids in ceramides, thus forming ω-*O*-acylceramide, a structural lipid that is essential for skin barrier function [14]. Fatty acids have also been proposed to be important for SC acidification [15].

**Fig. 1:**
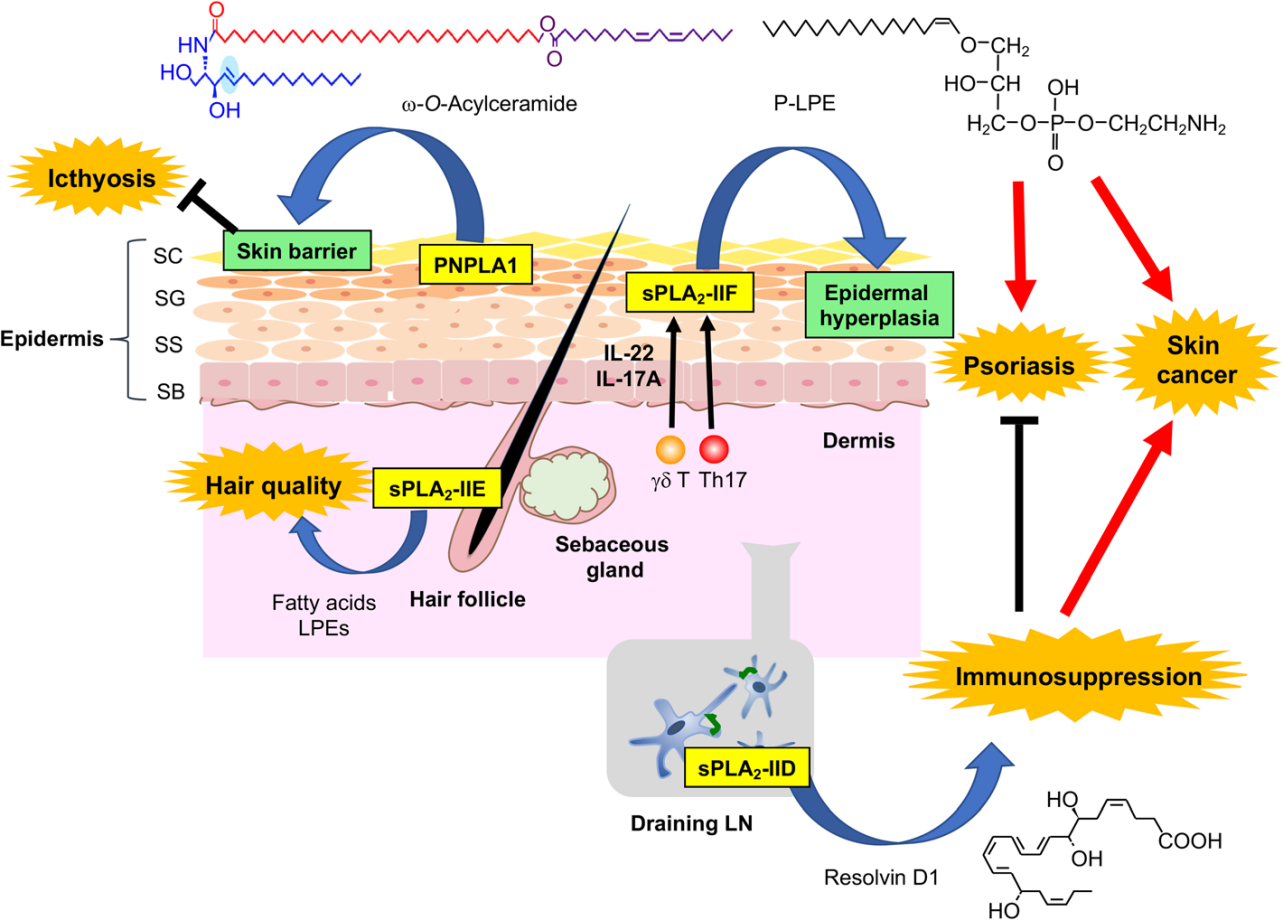
Expressions and functions of various PLA_2_s in mouse skin. sPLA_2_-IIF is localized in the suprabasal epidermis and produces P-LPE, which promotes epidermal hyperplasic diseases such as psoriasis and skin cancer. Epidermal sPLA_2_-IIF expression and thereby P-LPE production are augmented by IL-17A and IL-22 supplied by γδ T and Th17 cells in psoriasis. sPLA_2_-IIE is localized in hair follicles in synchrony with the growing phase (anagen) of hair cycling and may regulate hair homeostasis. sPLA_2_-IID is constitutively expressed in DCs and M2 macrophages in regional LNs and produces ω3 PUFA-derived anti-inflammatory lipid mediators, which put a brake on Th1 or Th17 immunity, thereby sequestering CHS and psoriasis and promoting skin cancer. PNPLA1 is expressed in the border of SG and SC, where it produces ω-*O*-acylceramide that is essential for skin barrier function. For details, please see the text

Release of fatty acids and lysophospholipids from glycerophospholipids (phospholipids hereafter) is catalyzed by phospholipase A_2_ (PLA_2_) enzymes, which are classified into several families as shown in Table 1 [16]. Until recently, however, it has remained obscure as to which PLA_2_ subtype(s) is important in the skin, which lipid species serve as the substrates and products for the PLA_2_(s), and how the PLA_2_-driven lipid metabolites affect skin pathophysiology. In this review, we highlight the distinct roles of several secreted PLA_2_s (sPLA_2_s) and the patatin-like phospholipase PNPLA1, whose functions have been revealed by recent studies using gene-manipulated (transgenic and knockout) mice in combination with mass spectrometry-based analytical techniques referred to collectively as lipidomics. Importantly, these enzymes are linked to unique lipid pathways distinct from canonical AA metabolism. The localizations and functions of particular PLA_2_s in the skin, as described in this review, are summarized in Fig. 1.

**Table 1:** The classification of PLA_2_ family. sPLA_2_, cPLA_2_, and iPLA_2_/PNPLA are the original big three among the PLA_2_ family. The sPLA_2_ family contains 10 catalytically active isoforms (IB, IIA, IIC, IID, IIE, IIF, III, V, X, XIIA) and 1 inactive isoform (XIIB) in mammals. The cPLA_2_ family comprises 6 isoforms (α-ζ). The human genome encodes 9 iPLA_2_ enzymes. These enzymes are now more generally referred to as PNPLA (1–9). In this review, the biological roles of particular PLA_2_s in the context of skin homeostasis and diseases were described

Family	Isoforms	Gene name	Common name	Alias	Enzyme property
sPLA_2_	11	PLA2G1B	sPLA_2_-IB	Pancreatic sPLA_2_	PLA_2_
		PLA2G2A	sPLA_2_-IIA	Non-pancreatic sPLA_2_	PLA_2_
		PLA2G2C	sPLA_2_-IIC		PLA_2_
		PLA2G2D	sPLA_2_-IID		PLA_2_
		PLA2G2E	sPLA_2_-IIE		PLA_2_
		PLA2G2F	sPLA_2_-IIF		PLA_2_
		PLA2G3	sPLA_2_-III		PLA_2_
		PLA2G5	sPLA_2_-V		PLA_2_
		PLA2G10	sPLA_2_-X		PLA_2_
		PLA2G12A	sPLA_2_-XIIA		PLA_2_
		PLA2G12B	sPLA_2_-XIIB		Inactive
cPLA_2_	6	PLA2G4A	cPLA_2_α		PLA_2_
		PLA2G4B	cPLA_2_β		PLA_1_/A_2_
		PLA2G4C	cPLA_2_γ		PLA_2_, transacylase, lysopholipase
		PLA2G4D	cPLA_2_δ		PLA_1_/A_2_
		PLA2G4E	cPLA_2_ε		PLA_1_/A_2_, *N-*acyltransferase
		PLA2G4F	cPLA_2_ζ		PLA_1_/A_2_
iPLA_2_/PNPLA	9	PNPLA1			Transacylase
		PNPLA2	iPLA_2_ζ	ATGL	TG lipase
		PNPLA3	iPLA_2_ε	ADPN	TG lipase, acyltransferase, restiny-esteryl lipase
		PNPLA4	iPLA_2_η	GS2	Retinyl-esteryl lipase?
		PNPLA5		GS2L	TG lipase
		PNPLA6	iPLA_2_δ	NTE	Lysophospholipase
		PNPLA7		NRE	Lysophospholipase
		PNPLA8	iPLA_2_γ		PLA_1_/A_2_
		PNPLA6	iPLA_2_β	PNPLA9	PLA_2_

## sPLA_2_-IIF, an epidermal sPLA_2_

The sPLA_2_ family consists of 11 isoforms with distinct substrate specificities and tissue distributions [17, 18]. Historically, several sPLA_2_s have been detected in mouse and human skin, but by using semi-quantitative RT-PCR and immunoblotting which have uncertain specificity [19–23]. sPLA_2_s have also been suggested to supply fatty acids for formation of the SC acid mantle, a hypothesis that stems primarily from the observation that SC acidity is perturbed by non-specific sPLA_2_ inhibitors [15, 23–25]. However, the molecular identity of any particular sPLA_2_(s) that participates in skin homeostasis and diseases has remained unclear until recently. Now, it has become obvious that sPLA_2_-IIF is a bona fide “epidermal sPLA_2_” that controls keratinocyte differentiation, hyperproliferation, and function [26].

Among the group II subfamily sPLA_2_s (which include sPLA_2_-IIA, sPLA_2_-IIC, sPLA_2_-IID, sPLA_2_-IIE, sPLA_2_-IIF, and sPLA_2_-V), sPLA_2_-IIF has several unique features [27, 28]. sPLA_2_-IIF has a uniquely long C-terminal extension that is proline-rich and contains a single cysteine, which raises the possibility that it might form a covalent homodimer, although this hypothesis has not been confirmed. In contrast to other group II subfamily sPLA_2_s that are basic proteins and catalytically active at neutral to mildly basic pH, sPLA_2_-IIF is an acidic protein (pI ~ 5.8) and retains its full enzymatic activity even at mildly acidic pH. This property may be related to the distribution of this enzyme in the upper epidermis (see below), which has a mildly acidic environment [15]. Furthermore, sPLA_2_-IIF is more hydrophobic than other sPLA_2_s, and probably because of this, it has a unique ability to penetrate and disrupt lipid monolayers and bilayers in vitro; when added exogenously, it rapidly enters the cells in an endocytosis-independent manner to form unusual aggregates [29]. Moreover, when overexpressed, sPLA_2_-IIF also tends to aggregate within the cells and can undergo *N*-glycosylation at three positions, possibly increasing its water solubility and thereby decreasing the unusual accumulation of sPLA_2_-IIF aggregates. However, it remains unknown whether or not endogenous sPLA_2_-IIF (or any other sPLA_2_s) is *N*-glycosylated in vivo. In a PLA_2_ enzyme assay using a phospholipid mixture extracted from mouse skin as a substrate (natural membrane assay [30]), a physiologically relevant concentration of sPLA_2_-IIF preferentially hydrolyzes phosphatidylethanolamine (PE; particularly plasmalogen-type PE) containing PUFAs (particularly DHA) to yield plasmalogen-type lysoPE (P-LPE) and DHA in preference to AA [26]. Therefore, although sPLA_2_-IIF is capable of releasing AA when overexpressed in mammalian cells at super-physiological levels [31], it may mobilize lipid metabolites separately from canonical AA metabolism under physiological conditions (see below).

**Fig. 2:**
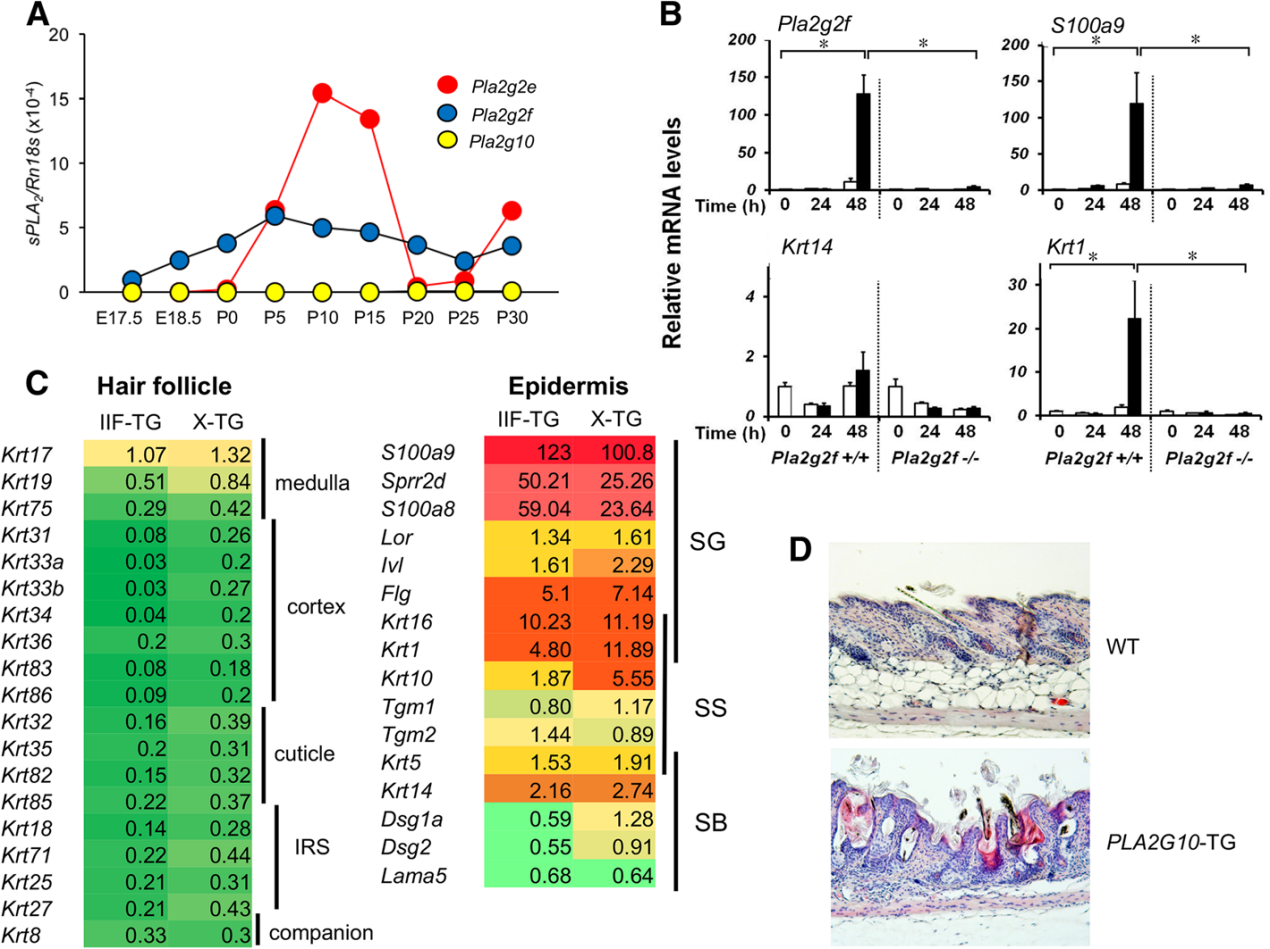
Skin abnormalities in knockout and transgenic mice for various sPLA_2_s. **a** Developmental expression of sPLA_2_s in mouse skin as assessed by quantitative RT-PCR. *Pla2g2f* is expressed throughout the peri- to postnatal period, while the periodic pattern of *Pla2g2e* expression coincides with the hair cycle, which involves repeated cycles of growth (anagen; P0–15), regression (catagen; P15–20), rest (telogen; P20–25), and re-growth (the next anagen; beyond P25). A representative result of two independent experiments is shown. **b** Expression of several keratinocyte markers in *Pla2g2f*^+/+^ and *Pla2g2f*^−/−^ keratinocytes cultured for the indicated periods with 1 mM Ca^2+^ (*n* = 4, mean ± SEM, *P < 0.05). *Pla2g2f* deficiency impairs the induction of *S100a9* (an activation marker) and *Krt1* (an SS marker), but not *Krt14* (a SB marker), suggesting that sPLA_2_-IIF regulates keratinocyte differentiation and activation. **c** Microarray profiling (Agilent Technologies) of genes associated with hair follicles and epidermis in *Pla2g2f*-TG (IIF-TG) or *PLA2G10*-TG (X-TG) mice relative to WT mice. In both strains, similar sets of genes are decreased in hair follicles (green), which reflects alopecia, and increased in the epidermis (red), which reflects epidermal hyperplasia. **d** Hematoxylin and eosin staining of skins from WT and *PLA2G10*-TG mice at P25. Hair follicle distortion and epidermal thickening are evident in the TG mice. IRS, inner root sheath. All animal experiments were performed in accordance with the protocols approved by the Institutional Animal Care and Use Committees in accordance with the Japanese Guide for the Care and Use of Laboratory Animals

It is now obvious that sPLA_2_-IIF is a major sPLA_2_ expressed in mouse epidermis, where it is distributed in the suprabasal SS, SG, and SC layers [26]. Developmental expression of *Pla2g2f* in mouse skin is far greater than that of other sPLA_2_s (except for *Pla2g2e*, see below), gradually increasing before birth to reach a maximum level by P5 (Fig. 2a). sPLA_2_-IIF expression is markedly induced during Ca^2+^-induced differentiation and also robustly upregulated in primary keratinocytes following stimulation with the Th17 cytokines IL-22 and IL-17A. Moreover, sPLA_2_-IIF is induced in mouse skin treated with imiquiod, an inducer of experimental psoriasis, and also highly expressed in the hyperplasic epidermis of patients with psoriasis. Strikingly, global or skin-specific transgenic mice overexpressing mouse sPLA_2_-IIF (*Pla2g2f*-TG) spontaneously develop psoriasis-like epidermal hyperplasia and alopecia, with increased expression of various psoriasis markers such as S100A9 and IL-36α [26], suggesting that increased expression of this sPLA_2_ alone could trigger psoriasis.

In a basal state, *Pla2g2f*^−/−^ mice have mild abnormalities in the skin (particularly the abdominal skin, probably because it is continuously exposed to friction against the ground surface), as revealed by a fragile stratum corneum with modest perturbation of skin barrier function and acidity [26]. After tape stripping of the SC, *Pla2g2f*^−/−^ mice display delayed recovery from the epidermal barrier perturbation [23]. In primary culture, keratinocytes from *Pla2g2f*^−/−^ mice fail to differentiate and become properly activated (Fig. 2b), and similar defects are evident when WT keratinocytes are treated with a pan-sPLA_2_ inhibitor or an sPLA_2_-IIF-directed siRNA. Most importantly, in pathological settings, *Pla2g2f*^−/−^ mice are protected from epidermal hyperplasia and associated inflammation in models of Th17-dependent psoriasis and Th1-dependent contact hypersensitivity (CHS) [26]. Consistent with this, *Pla2g2f* deficiency in keratinocytes markedly impairs the induction of several psoriasis markers in response to IL-17A or IL-22. Moreover, *Pla2g2f*^−/−^ mice are also protected from skin carcinogenesis, whereas *Pla2g2f*-TG mice conversely develop larger skin tumors than WT mice [26]. Mechanistically, sPLA_2_-IIF preferentially hydrolyzes plasmalogen-type PE secreted from keratinocytes to yield P-LPE, a unique lysophospholipid that facilitates the differentiation and activation of keratinocytes, leading to exacerbation of epidermal hyperplasia and inflammation. Indeed, the skin levels of P-LPE are correlated well with those of sPLA_2_-IIF expression in several skin disease models, and topical application of P-LPE to *Pla2g2f*^−/−^ skin in vivo or supplementation of *Pla2g2f*^−/−^ keratinocytes with P-LPE ex vivo restores the psoriasis-related phenotypes.

Taken together, these results indicate that sPLA_2_-IIF promotes epidermal hyperplasic diseases including psoriasis and skin cancer and that P-LPE, a primary sPLA_2_-IIF product, represents a biomarker and bioactive lipid that reflects the expression and function of sPLA_2_-IIF. Given that sPLA_2_-IIF is expressed in the epidermis rather specifically and that *Pla2g2f*^−/−^ mice display more profound skin phenotypes in diseases than in homeostasis, inhibition of this particular sPLA_2_ may be useful for treatment of psoriasis, skin cancer, or other conditions involving epidermal hyperplasia. It remains to be determined, however, whether sPLA_2_-IIF-driven P-LPE would act on keratinocytes through a specific receptor or through other mechanism(s). It is also possible that DHA, another sPLA_2_-IIF-driven product, would be metabolized to certain metabolites that could affect skin homeostasis, since DHA or its pro-resolving metabolites can facilitate skin wound healing, suppress psoriasis, and prevent neoplastic transformation of keratinocytes [32–34].

## sPLA_2_-IIE, a hair follicular sPLA_2_

Although sPLA_2_-IIE is not substantially expressed in the epidermis, it is a major “hair follicular sPLA_2_” in mice, being expressed in hair follicles in synchrony with hair cycling [35]. Thus, during the anagen phase, sPLA_2_-IIE is distributed in companion cells of the outer root sheath and cuticular cells of the inner root sheath in growing hair follicles. At P10–15, when hair follicles are maximally developed in the initial hair cycle, the expression of sPLA_2_-IIE becomes maximal, even exceeding that of sPLA_2_-IIF in the whole mouse skin (Fig. 2a). In contrast, during the catagen to telogen phase, when hair follicles regress, sPLA_2_-IIE expression promptly decreases to a negligible level, and then rises again in correlation with entry into the next anagen.

*Pla2g2e*^−/−^ mice exhibit mild skin abnormalities with perturbation of hair follicle ultrastructure and modest changes in the steady-state expression of a subset of skin genes. Lipidomics analysis has revealed that sPLA_2_-IIE mobilizes various unsaturated fatty acids and LPE species (both acyl and plasmalogen forms) in mouse skin, in agreement with the in vitro substrate selectivity of this enzyme [35]. Although several lipid mediators such as PGD_2_ and LPA play crucial roles in hair homeostasis [13, 36], the hair phenotypes observed in mice lacking sPLA_2_-IIE appear to be much milder than those in mice lacking synthases or receptors for these lipid mediators. Therefore, it still remains unclear which lipid metabolites mobilized by sPLA_2_-IIE are involved in the regulation of hair follicle homeostasis. Notably, in contrast to *Pla2g2f*^−/−^ mice, *Pla2g2e*^−/−^ mice do not exhibit psoriasis-related phenotypes [35], implying that these two skin sPLA_2_s—hair follicular sPLA_2_-IIE and epidermal sPLA_2_-IIF—play non-redundant roles in distinct compartments of mouse skin, underscoring the functional diversity of multiple sPLA_2_s in the coordinated regulation of skin homeostasis and diseases.

Since humans are essentially furless, it is unclear whether sPLA_2_-IIE is also expressed and plays certain roles in human hair follicles. It should be noted that although sPLA_2_-IIE expression is induced in several mouse tissues during inflammation [37], it is hardly detected in human tissues, representing a notable species difference. As sPLA_2_-IIA, the closest homolog of sPLA_2_-IIE, is highly induced during inflammation in humans [37], it has been proposed that the functions of sPLA_2_-IIA in humans might be compensated by sPLA_2_-IIE in mice [38].

## sPLA_2_-IID, a resolving sPLA_2_

While sPLA_2_-IIF and sPLA_2_-IIE are abundantly expressed in keratinocytes of the upper epidermis and hair follicles, respectively (see above), sPLA_2_-IID is barely detectable in mouse skin. Instead, sPLA_2_-IID is abundantly expressed in dendritic cells (DCs) and M2-like macrophages in secondary lymphoid organs such as the spleen and lymph nodes (LNs) of mice and humans [39, 40]. The expression of sPLA_2_-IID is downregulated, rather than upregulated, following inflammatory stimuli [39, 41]. This property is unique among the sPLA_2_ isoforms and probably reflects its role as a “resolving sPLA_2_” that counteracts inflammation [18, 39]. Despite the low expression of sPLA_2_-IID in skin, *Pla2g2d* deficiency leads to exacerbation of CHS and psoriasis. This is most likely because sPLA_2_-IID attenuates adaptive immunity in the LNs, thereby sequestering Th1 and Th17 immune responses [39, 40].

In a model of CHS, the resolution of inflammation in the skin and regional LNs is delayed in *Pla2g2d*^−/−^ mice [39]. In this state, expression of the Th1 cytokines IFN-γ and IL-12 is robustly elevated in the LNs. Likewise, in a model of psoriasis, *Pla2g2d*^−/−^ mice display more severe epidermal hyperplasia than do *Pla2g2d*^+/+^ mice, with increased IL-17A^+^ or IL-22^+^ T cells in the affected skin and LNs [40]. Furthermore, DCs isolated from *Pla2g2d*^−/−^ mice are hyper-activated even in the absence of stimulation. Mechanistically, sPLA_2_-IID in the LNs constitutively hydrolyzes PUFA-containing PE species (likely in microparticle membranes) to mobilize ω3 PUFA-derived anti-inflammatory lipid mediators, which can put a brake on DC-committed adaptive immunity. Indeed, the steady-state levels of ω3 PUFAs and their metabolites, such as DHA-derived resolvin D1 (RvD1), are markedly reduced in the LNs of *Pla2g2d*^−/−^ mice relative to *Pla2g2d*^+/+^ mice. Conversely, *Pla2g2d*-TG mice display milder inflammation in the CHS and psoriasis models, with increased levels of ω3 PUFA metabolites [40]. ω3 PUFA-derived resolvins and maresins suppress acquired immunity by attenuating migration and activation of DCs, antigen presentation to T cells, and IgE class switching in B cells [10, 39, 42, 43]. Moreover, these ω3 PUFA-derived lipid mediators have the ability to facilitate the polarization of anti-inflammatory M2 macrophages [44, 45], consistent with the fact that fewer M2 macrophages are present in the LNs of *Pla2g2d*^−/−^ mice [40].

On the other hand, the beneficial role of sPLA_2_-IID in counteracting harmful Th1/Th17 immune responses can be conversely disadvantageous in some situations such as host defense against infection and cancer [40, 46]. Indeed, sPLA_2_-IID promotes, rather than prevents, the development of skin tumors, likely because it attenuates anti-tumor Th1 immunity. Accordingly, *Pla2g2d*^−/−^ mice are protected against skin carcinogenesis, with increased numbers of tumor-suppressing cytotoxic T cells and M1 macrophages [40]. Thus, the immunosuppressive function of sPLA_2_-IID provides “good” or “bad” outcomes in distinct disease settings, protecting against skin inflammation and exacerbating skin cancer. In the latter context, specific inhibition of sPLA_2_-IID in patients with certain types of cancer would be a potentially attractive therapeutic intervention for restoration of immunological functions, a concept reminiscent of “immune checkpoint” therapy.

## Recalling sPLA_2_-IIA and sPLA_2_-X: a matter of species difference

As in the case of transgenic mice overexpressing sPLA_2_-IIF [26], those overexpressing human sPLA_2_-IIA or sPLA_2_-X (*PLA2G2A*-TG and *PLA2G10*-TG, respectively) also develop alopecia and epidermal hyperplasia, accompanied by cyst formation, sebaceous gland hyperplasia, and a disturbed hair stem cell fate (Fig. 2c, d) [47–49]. However, since neither sPLA_2_-IIA nor sPLA_2_-X is endogenously detected in mouse skin at a substantial level [26, 50], the intrinsic roles of these two sPLA_2_s in the skin have remained elusive. The discovery of sPLA_2_-IIF as a bona fide “epidermal sPLA_2_” in mice [26] has led to speculation that the skin phenotypes observed in *PLA2G2A*-TG or *PLA2G10*-TG mice may reflect the fact that sPLA_2_-IIA or sPLA_2_-X mimics the intrinsic actions of sPLA_2_-IIF when artificially overexpressed in the skin, or that endogenous sPLA_2_-IIF is upregulated in the hyperplasic epidermis of these transgenic mice. In support of the latter idea, the skin of *PLA2G10*-TG mice has elevated expression of sPLA_2_-IIF, with increased hydrolysis of DHA-containing PE species [26, 49], and microarray gene profiling of the skin reveals similar changes in gene expression between *PLA2G2F*-TG and *PLA2G10*-TG mice (Fig. 2c).

**Fig. 3:**
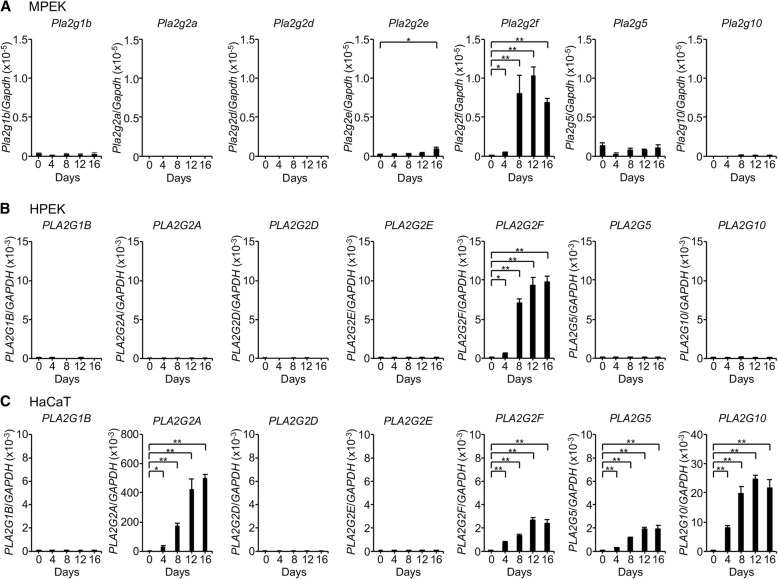
Expression of sPLA_2_s in mouse and human keratinocytes. Quantitative RT-PCR of various sPLA_2_s in MPEKs (**a**), HPEKs (**b**), and HaCaT cells (**c**) that were cultured for the indicated periods with 1 mM Ca^2+^ (*n* = 4, mean ± SEM, *P < 0.05). *PLA2G2F* is the dominant sPLA_2_ expressed in MPEKs and HPEKs, whereas *PLA2G2A* > *PLA2G10* > *PLA2G2F* > *PLA2G5* are expressed in HaCaT cells

However, considering the species difference between mice and humans, as already pointed out for the relationship between sPLA_2_-IIA and sPLA_2_-IIE (see above), it seems important to reconcile the expression of sPLA_2_-IIA or sPLA_2_-X in human keratinocytes. Indeed, beyond the uncertainty regarding the specificity of the detection methods employed, previous studies have demonstrated the expression of various sPLA_2_s in human keratinocytes [21]. Moreover, under the assumption that sPLA_2_-X is expressed in keratinocytes, exogenously added sPLA_2_-X can stimulate dendricity and pigmentation of human melanocytes through a mechanism dependent upon lysophosphatidylcholine [51]. We therefore reevaluated the expression of sPLA_2_s in human keratinocytes by quantitative RT-PCR. As in mouse primary epidermal keratinocytes (MPEKs) (Fig. 3a), *PLA2G2F* was induced following Ca^2+^-induced differentiation, whereas other sPLA_2_s including *PLA2G1B*, *PLA2G2A*, *PLA2G2D*, *PLA2G2E*, *PLA2G5*, and *PLA2G10* were barely detectable, in human primary epidermal keratinocytes (HPEKs) (Fig. 3b). In contrast, in the transformed human keratinocyte cell line HaCaT, there was robust Ca^2+^-induced upregulation of *PLA2G2A* and *PLA2G10*, which was even greater than that of *PLA2G2F* as well as *PLA2G5* (Fig. 3c). These results suggest that not only sPLA_2_-IIF, but also sPLA_2_-IIA, sPLA_2_-X, and possibly sPLA_2_-V can be expressed in transformed, rather than normal, human keratinocytes. Thus, although it is possible that sPLA_2_-IIA and sPLA_2_-X might participate in certain forms of skin pathology such as cancer, it is nonetheless likely that sPLA_2_-IIF is the primary sPLA_2_ acting in the epidermis of both mice and humans under physiological conditions. This is reminiscent of the fact that sPLA_2_-V is upregulated in the transformed mouse macrophage cell line P388D_1_ [52], whereas it is not induced, but rather downregulated, in primary mouse macrophages [38], after stimulation with LPS or zymosan. Therefore, caution should be exercised when interpreting the data obtained from studies using transformed cell lines.

## PNPLA1, an ω-*O*-acylceramide synthase (transacylase)

The epidermis contains a unique class of ceramides with ω-hydroxy ultra-long chain fatty acids (C30–C36) esterified specifically with LA. This particular ceramide class is called ω-*O*-acylceramide, a key lipid component essential for skin barrier function [53]. The unique structure and high hydrophobicity of ω-*O*-acylceramide are important for the organization and function of lipid lamellae in the SC, where this unique lipid serves as a “molecular rivet” that connects adjacent lamellar membrane structures. ω-*O*-acylceramide also acts as a precursor of protein-bound ceramides for formation of the cornified lipid envelope, where a lipid monolayer is covalently bound to the cornified envelope. A series of recent studies on patients with congenital ichthyosis have revealed that many of the causal genes are related to the biosynthesis and metabolism of ω-*O*-acylceramide [54]. The entire picture of ω-*O*-acylceramide metabolism has been comprehensively summarized in other recent reviews [14, 55].

A recent breakthrough in this research area has been the identification of PNPLA1, a member of the iPLA_2_ family, as a long-sought ω-*O*-acylceramide synthase, whose genetic mutations in humans and dogs cause congenital ichthyosis [56] and deletion in mice leads to neonatal death due to excessive transepidermal dehydration resulting from severe skin barrier defect [57–59]. PNPLA1 catalyzes the unique transacylase reaction, whereby the LA moiety cleaved from triacylglycerol through the lipase-like reaction of this enzyme is directly transferred to the ω-hydroxy moiety of ultra-long chain fatty acid in ceramide (ω-*O*-hydroxyceramide), with the ω-hydroxy group, instead of water, serving as an acyl (linoleoyl) acceptor [60]. Thus, on the basis of PLA_2_ biology, PNPLA1 is particularly unique in that it (i) is involved in the metabolism of sphingolipids rather than glycerophospholipids, (ii) catalyzes transacylation rather than hydrolysis of target substrates, and (iii) recognizes the specific lipoquality of LA and ultra-long chain fatty acids.

Of additional note, PLA2G15 (also known as lysosomal PLA_2_ or LPLA2) has the capacity to catalyze the biosynthesis of 1-*O*-acylceramide through transacylation of fatty acid from the *sn*-2 position of phospholipid to the 1-hydroxy group of ceramide [61]. 1-*O*-acylceramide is a natural component of human and mouse epidermis [62]. However, the biological importance of this unique lipid and the contribution of PLA2G15 to its biosynthesis in vivo are unclear.

## Conclusions

Healthy skin depends on a unique lipid profile to form a barrier that confers protection and prevents excessive water loss, aids cell-cell communication, and regulates cutaneous homoeostasis and inflammation. Alterations in the cutaneous lipid profile often have severe consequences for skin health and have been implicated in various skin diseases. Recent developments in lipidomics technologies now allow in-depth qualitative and quantitative investigation of a wide variety of cutaneous lipids, providing insight into their roles and mechanistic actions [63]. Cross-communication between various types of bioactive lipids suggests that their cutaneous activities should be considered as part of a wider metabolic network that can be targeted to maintain skin health, control inflammation, and improve skin pathologies [64].

Given that PLA_2_s are crucial enzymes for the control of lipoquality, it is of particular importance to understand the expression and function of each PLA_2_ in a specific skin niche. In addition to sPLA_2_s and PNPLA1, which we have focused on here, several biochemical and pharmacological studies have suggested potential contributions of other PLA_2_s such as cytosolic PLA_2_s (cPLA_2_α and cPLA_2_δ) to skin inflammation [65–68], although these findings should be confirmed by genetic studies using knockout mice for these enzymes. Our preliminary study has revealed that several other PLA_2_s are also expressed in different cell populations and may play distinct roles in skin homeostasis and inflammation. Thus, unveiling the entire view of lipid metabolism driven by various forms of PLA_2_s will support translational studies exploring the involvement of lipids in skin health and disease.

## Abbreviations

AA: Arachidonic acid
CHS: Contact hypersensitivity
DC: Dendritic cells
DHA: Docosahexaenoic acid
EPA: Eicosapentaenoic acid
HPEKs: Human primary epidermal keratinocytes
iPLA_2_: Ca^2+^-independent phospholipase A_2_
LA: Linoleic acid
LN: Lymph nodes
LPA: Lysophosphatidic acid
LT: Leukotriene
MPEKs: Mouse primary epidermal keratinocytes
PE: Phosphatidylethanolamine
PG: Prostaglandin
P-LPE: Plasmalogen-type lysoPE
PNPLA: Patatin-like phospholipase
PUFA: Polyunsaturated fatty acid
Rv: Resolvin
SB: Stratum basale
SC: Stratum corneum
SG: Stratum granulosum
sPLA_2_: Secreted phospholipase A_2_
SS: Stratum spinosum
